# Association of RBP4 Genotype with Phenotypic Reproductive Traits of Sows

**DOI:** 10.1155/2016/4940532

**Published:** 2016-01-14

**Authors:** A. Marantidis, G. P. Laliotis, M. Avdi

**Affiliations:** Laboratory of Physiology of Reproduction of Farm Animals, Department of Animal Production, School of Agriculture, Aristotle University of Thessaloniki, 54124 Thessaloniki, Greece

## Abstract

PCR-RFLP was applied to a commercial crossbred pig population in order to investigate the association between polymorphism (SNP) of Retinol-binding protein 4 (RBP4) gene and reproductive performance. 400 sows were genotyped and 2000 records of reproductive traits were used in order to retrieve information about the allele frequencies and the association of the RBP4 gene with main reproductive characteristics of the population. A deviation from the Hardy-Weinberg equilibrium was observed as a result of the AB genotype excess. In addition, the AA genotype saw statistically significant higher values of (i) the total number of born piglets (*p* < 0.05), (ii) the number of piglets born alive (*p* < 0.01), and (iii) the number of weaned piglets (*p* < 0.01). The number of the mummified piglets and the number of the piglets born dead did not differ between the various RBP4 genotypes. Interestingly, the AA genotype had a negative impact (*p* < 0.05) on the number of piglets born dead, resulting indirectly in a larger litter size. In conclusion, the AA genotype and in extension the A allele of RBP4 gene are in favor of producing larger litter size, suggesting that the RBP4 gene may be used in Marker-Assisted Selection (MAS) programs for a rapid improvement of the reproductive characteristics in pigs.

## 1. Introduction

The implementation of reliable genetic markers on the Marker-Assisted Selection (MAS) programs applied to the pig industry may result in small to moderate increases in litter size that would improve farm economic performance. In addition, the early selection of the sows, before they reach reproductive maturity, would stop breeding low-performance sows [1]. An effective way to detect genetic markers is the candidate gene approach, where the identification of gene polymorphisms that cause variation in a trait is based on physiological, immunological, or endocrine evidence [2].

The RBP4 gene is expressed during the period of fast elongation of the pig blastocyst [3]. This is a critical period for the survival of the embryos. Harney et al. [4] reported an increased expression of the RBP4 gene in the gravid endometrium, between the 10th to 12th day of pregnancy of sows, suggesting that the respective coding protein (RBP4) plays an important role in uterine and conceptus physiology during the establishment of pregnancy. In addition, Retinol-binding protein 4 (RBP4) has been found to be a major secretory product of the pig conceptus prior to implantation [5]. Therefore, RBP4 is reported as a candidate gene for litter size owing to its possible role at the time of embryonic development [1].

An initial study in hyperprolific and control sows yielded an estimated additive effect (0.4 piglets/birth) of RBP4 gene [6]. In a follow-up study, Rothschild et al. [1] checked whether this effect was also detectable in commercial lines of pigs. They found an approximate additive effect of 0.23 pigs per litter (*p* < 0.05) for TNB (total number of born piglets) and 0.15 pigs per litter for NBA (number of piglets born alive) suggesting that RBP4 probably affects the litter size in some commercial lines. However, later studies in various crossbreds and pure breeds have not always confirmed these results. Even though the same trend was obtained in most cases, a statistically significant difference was not always observed [7, 8] or it was restricted to some parities [9, 10].

One of the major problems of the Greek pig industry is the low prolificacy performance reflecting a narrow economic income. To the best of our knowledge there is no previous reported study investigating any interaction between RBP4 gene polymorphism and reproductive traits in Greek pig farming, as a tool of identification of the more productive animals. Therefore, the aim of this study was to determine any possible associations of the RBP4 genotypes with main reproductive traits of sows in a commercial population reared in Greece, so as to enable such information to be used in the future for breeding selection schemes.

## 2. Materials and Methods

### 2.1. Animals and Data Collection

The pig population was derived from a Greek commercial farm (North Western of Greece). 400 sows in total (crossbreed from Large White × Landrace) were genotyped. The sows were randomly selected from the whole population of farm among those who gave their first birth at the age of 12 months and had at least 5 continuous litters. Sows were artificially inseminated with fresh semen derived from Duroc × Pietrain boars. All sows were kept under the same feeding and housing conditions and their reproductive performance was permanently recorded by the staff of the farm. Reproductive traits that were taken into account were (i) the total number of born piglets (TNB), (ii) the number of piglets born alive (NBA), (iii) the number of piglets born dead (NBD), (iv) the number of mummified piglets (NBM), (v) the number of aborted piglets (ABRT), and (vi) the number of weaned piglets (NW).

### 2.2. DNA Isolation and Genotyping

Polymerase chain reaction-restriction fragment length polymorphism (PCR-RFLP) for the RBP4 gene genotyping was performed. DNA was extracted from hair roots or blood of the sows, using the Nucleospin blood or tissue kits (Macherey-Nagel, Germany). An electrophoresis was performed to ensure the integrity of the DNA samples. Concerning the PCR procedure, the primer sequences used were 5′-GAGCAAGATGGAATGGGTT-3′ and 5′-CTCGGTGTCTGTAAAGGTG-3′ for the forward and the reverse primer, respectively [1]. The PCR amplification was performed as follows: approximately 150 ng of genomic DNA was used as template and amplified in a final volume of 25 *μ*L containing 200 nM of each primer, 1 mM dNTPs, and 1 unit MyTaq DNA Polymerase (Bioline). PCR amplification was performed using the following conditions: initial denaturation at 95°C for 5 min, 30 amplification cycles including denaturation at 95°C for 30 s, annealing at 56°C for 30 s, and extension at 72°C for 45 s and a final extension step at 72°C for 10 min. Finally, 15 *μ*L of PCR product (550 bp) was digested in a total volume of 20 *μ*L, containing 10 U of enzyme MspI (Takara), 2 *μ*L of restriction buffer, and 2 *μ*L of BSA for 3 hours. Restriction fragments were examined by electrophoresis on 2.5% agarose gel with 1x TBE buffer.

### 2.3. Statistical Analysis

Genotype frequencies, allele frequencies, and Hardy-Weinberg equilibrium estimations were calculated using PopGene Software v. 1.32 [11]. The statistical procedures were performed using the SPSS program (version 19.0). A mixed statistical model was used for the analysis of associations between the RBP4 genotypes and the total number of born piglets (TNB), the number of piglets born alive (NBA), the number of piglets born dead (NBD), the number of piglets born mummified (BMUM), the number of aborted piglets (ABRT), and the number of weaned piglets (NW). Due to the uneven distribution of the first litters, their parameters were analyzed separately. The statistical models used in the analysis were as follows:

(1) $$\begin{array}{c} Y_{ijk}=\mu +G_{i}+L_{j}+\left(\;G_{i}*L_{j}\;\right)+T_{k}+e_{ijk}, \end{array}$$

where *Y*_*ijk*_ is trait value, *μ* is the general mean, *G*_*i*_ is the fixed effect of RBP4 genotype (*i* = 1,2, 3), *L*_*j*_ is the fixed effect of the litter parity (*j* = 2,3, 4,5), *G*_*i*_ *∗L*_*j*_ is the effect of the interaction between the *i* genotype and the *j* litter parity, *T*_*k*_ is the random effect of the sow (*k* = 1,2,…, 400), and *e*_*ijk*_ is the random error.

When the data of the examined traits were analyzed for each parity separately, the factors regarding *L*_*j*_ (the fixed effect of litter parity) and *G*_*i*_ *∗L*_*j*_ (the effect of interaction between the *i* genotype and the *j* litter parity) were excluded from the model.

## 3. Results

### 3.1. Genotype and Allele Distribution of RBP4 Gene

Two RBP4 alleles (A, B) and three genotypes, namely, AA, AB, and BB, were identified in the examined population. The allelic and genotypic frequencies are presented in Table 1. Allele frequencies were 0.56 and 0.44 for allele A and allele B, respectively. A heterozygosity excess was observed, while the population was found to deviate (*p* < 0.05) from the Hardy-Weinberg equilibrium.

### 3.2. Association of Sows' Reproductive Traits and RBP4 Genotypes

The results of mean TNB, NBA, NBD, NBM, ABRT, and NW values (piglets/birth) in regard to the observed RBP4 genotypes are presented in Table 2. Statistically significant differences were detected between genotypes AA and AB as well as between AA and BB genotypes for almost all analyzed reproductive traits. Specifically, in regard to the TNB value, the AA genotype showed a higher (*p* < 0.05) number of piglets/litter (13.82 ± 0.10) compared to the AB (13.51 ± 0.07) and the BB (13.44 ± 0.13) genotype. The same significant (*p* < 0.01) trend was also observed for the number of born piglets (NBA) in reference to the respective RBP4 genotypes. Nonsignificant differences were observed for the number of piglets born mummified (BMUM) and the aborted piglets (ABRT) among the observed RBP4 genotypes. Interestingly, the AA genotype gave a statistically significant (*p* < 0.05) lower number of piglets born dead (NBD) per litter (0.54 ± 0.04) with respect to the two other analyzed genotypes. Moreover, the sows with the AA genotype had also larger number (*p* < 0.01) of piglets than the sows carrying the AB and the BB genotypes. Specifically, the AA genotype produced 0.52 weaned piglets (NW) more than the BB genotypic sows. This difference also remained statistically significant (*p* < 0.01) in the first parity (Table 3), with the AA genotype still having the largest litter size (10.47 ± 2.15) compared to the AB (10.03 ± 2.05) and the BB (10.15 ± 2.66) genotype. As far as it concerns TNB and NBA traits, it revealed that the AA and/or the AB genotypes are in favor of producing larger litter size in regard to the BB genotype among all analyzed parities (Table 3), except the first parity.

## 4. Discussion

Herein we reported the influence of the RBP4 gene on the prolificacy of a crossbreed population reared in Greece. 400 sows were genotyped and 2000 records were used in order to retrieve information about allele frequencies and the association of the RBP4 gene on main reproductive characteristics of the population.

According to our data, an excess of the AB genotype was observed and a higher frequency of allele A (0.56) with respect to allele B was noted. Similar allelic frequencies have been reported for Large White and Landrace × Large White populations [1, 7, 12–14] and in Black Slavonian sows [15]. Higher allelic values for A allele have been previously reported in Duroc [16] and in Polish Landrace [17] sows populations, while lower values have been reported by Kapelański et al. [18] in Polish Landrace and in Police Large White populations. The analyzed population was found to deviate from the Hardy-Weinberg equilibrium due to the higher value of heterozygous genotype, as also reported by Omelka et al. [19].

As far as it concerns the effect of RBP4 genotype on main reproductive traits of the studied population, it was revealed that both the AA and the AB genotypes were favored in producing statistically significant higher values of TNB, NBA, and NW traits (piglets/birth), rendering the A allele as an allele with an additive effect. Our results are in agreement with previous studies [2, 6, 20], which reported that the AA genotypes were associated with higher TNB and NBA piglets/birth. Moreover, previous authors [1, 9] concluded that the presence of B allele had a negative effect on the litter size, suggesting that A allele was in favor of prolificacy. In addition, Sun et al. [21] reported that crossbreed pigs with the AA genotype produced 0.72 TNB, 0.64 NBA more than the BB genotypic sows, while Gonçalves et al. [22] reported that A allele of RBP4 gene produced more piglets (TNB) and more live piglets per litter (NBA).

Contrary to our results, data obtained in other sows' populations (crossbreeds or pure breeds) failed to reach statistically significant difference in regard to the RBP4 genotypes and prolificacy [16, 23]. Furthermore, other researchers noted that BB genotypes displayed higher litter sizes than the AA and the AB genotypes [7, 24]. The B allele originates from a Chinese pig [25] and is associated also with high fertility performance [26]. The fact that in the studied population the B allele had a negative effect on the examined reproductive traits may reflect the absence of Chinese ancestors in our population.

It is worth noting that the AA genotype had also a negative impact (*p* < 0.05) on the number of piglets that were born dead, with respect to the other two genotypes (AB and BB) reflecting indirectly a greater litter size. To our knowledge this is the first time that a certain genotype is associated with the number of piglets that may be born dead in a litter.

Recent developments in the porcine genome maps set the basis for the identification of individual genes that affect reproduction. Therefore, the application of MAS on swine production may become more efficient as more associations between markers and traits are identified. This seems to be promising especially for litter size due to the low heritability and the sex limited nature of these traits [27]. Allele effects may differ between lines or populations due to the genetic background, rendering the establishment of a certain genotype expressing an improved reproductive trait not an easy task [15]. The A allele of RBP4 seems to impart an additive effect on the litter size rendering itself as a potential molecular marker in pig breeding schemes.

## 5. Conclusion

Our results on the RBP4 gene polymorphism studied in a commercial pig population showed that polymorphism of the RBP4 gene can be related to litter size. Statistical analysis revealed that sows with AA genotype had statistically higher litter sizes than those with BB genotypes, which displayed lower TNB, NBA, and NW values and higher NBD value. In addition, according to our results, A allele of the RBP4 gene seems to render an additive effect to the desired phenotypic reproductive traits (litter size), suggesting that this allele can be included in future Marker-Assisted Selection programs in sows' populations.

## Figures and Tables

**Table 1:** Genotype distribution and allele frequencies of the RBP4 gene in the analyzed population.

Genotype	Observed	Expected	Frequencies (*P*)
AA	113	125	0.28
AB	221	197	0.55
BB	66	78	0.17

*p* = 0.56; *q* = 0.44; *χ* ^2^ = 5.81; df = 1.

**Table 2:** Association of major reproductive traits with RBP4 genotypes among four parities (*N* = 400, 2–5 parities). The values presented as mean ± SEM^†^.

Genotype	TNB	NBA	NBD	NBM	ABRT	NW
AA	13.82 ± 0.10^a,*∗*^	13.02 ± 0.11^a,*∗∗*^	0.54 ± 0.04^a,*∗*^	0.25 ± 0.03^NS^	0.04 ± 0.10^NS^	12.30 ± 0.11^a,*∗∗*^
AB	13.51 ± 0.07^b,*∗*^	12.61 ± 0.08^b,*∗∗*^	0.64 ± 0.03^b,*∗*^	0.27 ± 0.02^NS^	0.05 ± 0.07^NS^	11.91 ± 0.08^b,*∗∗*^
BB	13.44 ± 0.13^b,*∗*^	12.55 ± 0.14^b,*∗∗*^	0.60 ± 0.05^b,*∗*^	0.29 ± 0.04^NS^	0.03 ± 0.01^NS^	11.78 ± 0.14^b,*∗∗*^

^†^TNB: total number of born piglets; NBA: number of piglets born alive; NBD: number of piglets born dead; NBM: number of mummified piglets; ABRT: number of aborted piglets; NW: number of weaned piglets. ^a,b^Different superscripts in the same column indicate significant difference (^*∗*^ *p* < 0.05; ^*∗∗*^ *p* < 0.01; ^NS^not significant).

**Table 3:** Genotype performance among the parities of 400 sows.

Parity	Genotype	*n*	TNB (mean + SD)	NBA (mean + SD)	NBD (mean + SD)	NBM (mean + SD)	ABRT (mean + SD)	NW (mean + SD)
1st	AA	113	11.73 ± 2.13	10.90 ± 2.18	0.56 ± 0.72	0.27 ± 0.67	0.04 ± 0.18	10.47 ± 2.15^a,*∗∗*^
	AB	221	11.52 ± 1.98	10.59 ± 2.10	0.66 ± 0.78	0.28 ± 0.62	0.05 ± 0.23	10.03 ± 2.05^b^
	BB	66	11.56 ± 2.21	10.48 ± 2.50	0.65 ± 1.30	0.42 ± 1.42	0.03 ± 0.17	10.15 ± 2.66^b^

2nd	AA	113	13.25 ± 2.00	12.58 ± 2.14	0.44 ± 0.61	0.22 ± 0.56	0.04 ± 0.18	11.85 ± 2.19^a,*∗*^
	AB	221	12.96 ± 2.04^a,*∗∗*^	12.15 ± 2.34^a,*∗∗*^	0.53 ± 0.70	0.27 ± 0.78	0.05 ± 0.23	11.39 ± 2.419^b,*∗*^
	BB	66	12.71 ± 2.38^b,*∗∗*^	11.97 ± 2.46^b,*∗∗*^	0.53 ± 0.98	0.21 ± 0.45	0.03 ± 0.17	11.29 ± 2.58^a,*∗∗*^

3rd	AA	113	14.04 ± 2.15^a,*∗*^	13.11 ± 2.22^a,*∗*^	0.69 ± 0.73	0.24 ± 0.51	0.04 ± 0.18	12.31 ± 2.29^a,*∗*^
	AB	221	13.51 ± 2.17^b,*∗*^	12.56 ± 2.26^b,*∗*^	0.69 ± 0.83	0.26 ± 0.52	0.05 ± 0.23	11.78 ± 2.29^b,*∗*^
	BB	66	13.53 ± 2.32^a,*∗*^	12.58 ± 2.42^a,*∗*^	0.55 ± 0.86	0.41 ± 0.66	0.03 ± 0.17	11.67 ± 2.46^a,*∗*^

4th	AA	113	14.12 ± 2.39	13.30 ± 2.59^a,*∗*^	0.54 ± 0.67	0.27 ± 0.52	0.04 ± 0.19	12.34 ± 2.55^a,*∗*^
	AB	221	13.8 ± 2.31	12.80 ± 2.40^b,*∗*^	0.72 ± 0.79	0.28 ± 0.53	0.05 ± 0.23	11.98 ± 2.44^b,*∗*^
	BB	66	13.98 ± 2.50	13.11 ± 2.61^a,*∗*^	0.62 ± 0.67	0.26 ± 0.62	0.03 ± 0.17	11.97 ± 2.74^a,*∗*^

5th	AA	113	13.87 ± 2.16^a,*∗*^	13.08 ± 2.09^a,*∗*^	0.50 ± 0.63	0.29 ± 0.66	0.04 ± 0.19	12.71 ± 1.95^a,*∗*^
	AB	221	13.76 ± 1.95^b,*∗*^	12.93 ± 1.76^b,*∗*^	0.60 ± 0.67	0.28 ± 0.52	0.05 ± 0.23	12.51 ± 1.73^b,*∗*^
	BB	66	13.52 ± 2.15	12.54 ± 2.12^a,*∗*^	0.71 ± 0.82	0.28 ± 0.55	0.03 ± .0.17	12.18 ± 1.97^a,*∗*^

^†^TNB: total number of born piglets; NBA: number of piglets born alive; NBD: number of piglets born dead; NBM: number of mummified piglets; ABRT: number of aborted piglets; NW: number of weaned piglets. ^a,b^Different superscripts in the same column and parity indicate significant difference (^*∗*^ *p* < 0.05; ^*∗∗*^ *p* < 0.01).
